# The proteome of the blood–brain barrier in rat and mouse: highly specific identification of proteins on the luminal surface of brain microvessels by in vivo glycocapture

**DOI:** 10.1186/s12987-024-00523-x

**Published:** 2024-03-04

**Authors:** Tammy-Lynn Tremblay, Wael Alata, Jacqueline Slinn, Ewa Baumann, Christie E. Delaney, Maria Moreno, Arsalan S. Haqqani, Danica B. Stanimirovic, Jennifer J. Hill

**Affiliations:** 1 Human Health Therapeutics, National Research Council Canada, 100 Sussex Dr., Ottawa, ON K1A 0R6 Canada; 2 Present Address: Biology Program, New York University Abu Dhabi, Saadiyat Island Campus, P.O. Box 129188, Abu Dhabi, United Arab Emirates

**Keywords:** Blood–brain barrier, Proteomics, Luminal, Endothelial, Vessel

## Abstract

**Background:**

The active transport of molecules into the brain from blood is regulated by receptors, transporters, and other cell surface proteins that are present on the luminal surface of endothelial cells at the blood–brain barrier (BBB). However, proteomic profiling of proteins present on the luminal endothelial cell surface of the BBB has proven challenging due to difficulty in labelling these proteins in a way that allows efficient purification of these relatively low abundance cell surface proteins.

**Methods:**

Here we describe a novel perfusion-based labelling workflow: in vivo glycocapture. This workflow relies on the oxidation of glycans present on the luminal vessel surface via perfusion of a mild oxidizing agent, followed by subsequent isolation of glycoproteins by covalent linkage of their oxidized glycans to hydrazide beads. Mass spectrometry-based identification of the isolated proteins enables high-confidence identification of endothelial cell surface proteins in rats and mice.

**Results:**

Using the developed workflow, 347 proteins were identified from the BBB in rat and 224 proteins in mouse, for a total of 395 proteins in both species combined. These proteins included many proteins with transporter activity (73 proteins), cell adhesion proteins (47 proteins), and transmembrane signal receptors (31 proteins). To identify proteins that are enriched in vessels relative to the entire brain, we established a vessel-enrichment score and showed that proteins with a high vessel-enrichment score are involved in vascular development functions, binding to integrins, and cell adhesion. Using publicly-available single-cell RNAseq data, we show that the proteins identified by in vivo glycocapture were more likely to be detected by scRNAseq in endothelial cells than in any other cell type. Furthermore, nearly 50% of the genes encoding cell-surface proteins that were detected by scRNAseq in endothelial cells were also identified by in vivo glycocapture.

**Conclusions:**

The proteins identified by in vivo glycocapture in this work represent the most complete and specific profiling of proteins on the luminal BBB surface to date. The identified proteins reflect possible targets for the development of antibodies to improve the crossing of therapeutic proteins into the brain and will contribute to our further understanding of BBB transport mechanisms.

**Supplementary Information:**

The online version contains supplementary material available at 10.1186/s12987-024-00523-x.

## Background

**Fig. 1:**
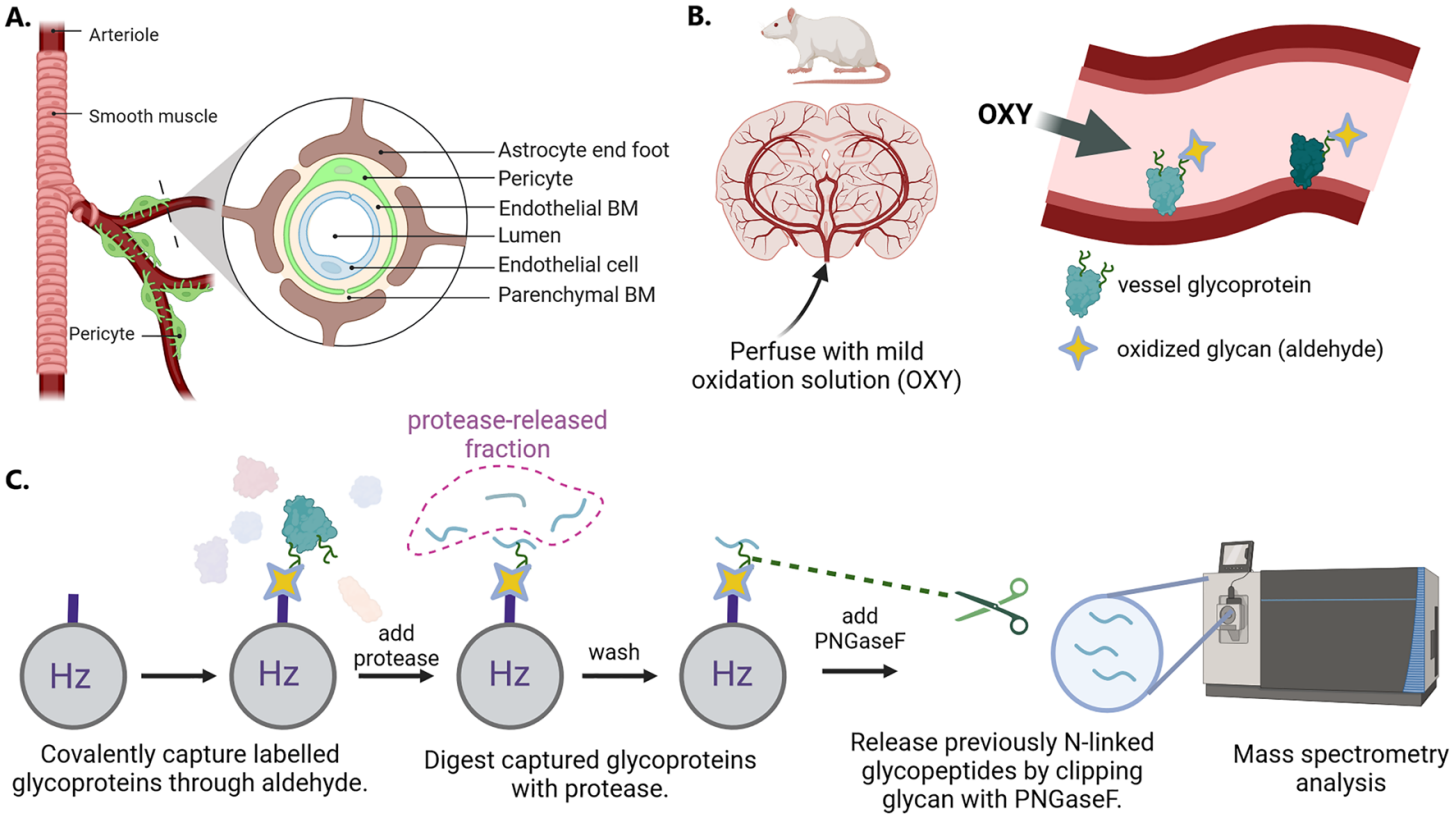
Overview of the in vivo glycocapture method for labelling luminal glycoproteins at the blood–brain barrier. **A** Depiction of the typical neurovascular unit at the BBB, showing the lumen of the blood vessel and its surrounding cells and basement membrane (BM). **B** Perfusion-based labelling of glycoproteins in rats and mice using a mild oxidation solution to form aldehydes on luminal glycans, including glycoproteins. **C** Glycocapture sample preparation workflow to specifically isolate peptides from proteins with oxidized glycans. A hydrazide (Hz) functionalized bead forms a covalent bond with aldehydes, capturing labelled glycoproteins and allowing unbound proteins to be washed away. Bound glycoproteins are then digested with a protease (trypsin, chymotrypsin) and unbound non-glycopeptides are washed away. Formerly N-glycosylated peptides are specifically released from the beads by the enzyme PNGase F and this collection of peptides is analyzed by mass spectrometry. *Created with BioRender.com*

The blood–brain barrier (BBB) is composed of a tightly organized subset of cells, often referred to as the neurovascular unit (NVU) that controls the transport of molecules from the blood into the brain [1–4]. The prototypical NVU consists of endothelial cells connected by tight junctions to form microvessels, where the luminal surface of the polarized endothelial cells is in contact with blood and the basal surface is coated by an acellular basement membrane comprised mainly of extracellular matrix proteins such as collagens and laminins (Fig. 1A). This vascular basement membrane layer is further surrounded by pericyte cells and can merge with a parenchymal basement membrane in areas without pericytes [5]. Outside of the parenchymal basement membrane, astrocyte endfeet surround the vessel and provide a connection to neurons in the brain parenchyma. The NVU is thought to be the cellular basis for the tight regulation of transport of nutrients, metabolites, and immune cell entry across the BBB and into the brain and central nervous system.

The luminal surface of endothelial cells is in direct contact with blood and includes a wide variety of transporter proteins that shuttle nutrients and metabolites across the BBB in a tightly-regulated manner via transcytosis [6]. The tight regulation of molecules crossing the BBB represents one of the largest challenges in the treatment of neurological diseases, such as Alzheimer’s and Parkinson’s disease, since many therapeutics are not able to enter the brain parenchyma due to their inability to cross the BBB efficiently, including nearly all protein-based drugs such as antibodies. One promising approach to facilitate trafficking of protein therapeutics into the brain is to use a “carrier” protein that binds to a transcytosing receptor present on the surface of the endothelial cells, allowing the therapeutic to piggyback on this receptor to gain access into the brain through receptor-mediated transcytosis (RMT) [7–10]. A thorough profiling of the proteins present at the BBB would improve our understanding of this unique physiological structure and the proteins responsible for trafficking through the BBB. Proteins present at the BBB also represent potential targets for development of therapeutics that can enable brain delivery.

Given the importance of the proteins present on the luminal surface of the BBB, several groups have focused on profiling these proteins using proteomic methods, as we have recently reviewed [11]. Proteomic analyses of isolated vessels have successfully identified many endothelial cell surface proteins, including known BBB transporter proteins [12–15]. However, these methods identify proteins from all cells present in the vessels and cannot determine the localization of these proteins, such as whether these proteins are present on the specialized luminal surface of the endothelial cells. To provide data on the proteins exposed to blood in brain vessels, chemical labelling has been used to specifically modify luminal proteins via perfusion of a reactive chemical label through animal brain capillaries [16–18]. All of these studies relied on perfusion of an N-hydroxysuccinimide (NHS)-ester modified biotin molecule that reacts with the N-terminus or exposed lysines on vessel-accessible proteins. Interestingly, significantly fewer proteins were identified using this approach in brain than from other organs, such as liver or kidney [11, 16, 18]. Furthermore, the specificity for cell surface proteins was generally low (~ 10–30%) in these approaches resulting in the identification of a very low number of cell surface membrane proteins. These findings suggest that labelling efficiency with biotin is much lower in the brain relative to other organs when relying on conjugation via NHS-esters.

One of the defining features of the BBB vasculature, relative to vasculature in other organs, is the particularly thick glycocalyx layer lining the luminal surface of the vessels [19]. As the name suggests, the glycocalyx is largely composed of glycans, in the form of glycoproteins, glycolipids, and proteoglycans. Given these two observations, we hypothesized that the glycan-based glycocalyx layer may be limiting the accessibility of luminal-facing proteins for reaction with NHS-esters. Therefore, since the majority of cell surface proteins are glycosylated, we decided to utilize a chemical labelling approach that targets glycans in the hopes of enriching luminal vessel proteins at the BBB in a more efficient and selective manner.

To this end, we modified a cell surface labelling approach which has been previously applied to isolated cells and tissues, called Cell Surface Capture (CSC) [20–22]. Unlike the NHS-biotinylation approach, which requires accessible primary amines (protein N-terminus or lysine residues), this cell surface labelling method specifically modifies glycans using mild oxidizing conditions. This oxidation reaction produces an aldehyde group which can then be used as a chemical “handle” for specific reaction with a hydrazide or aminooxy group. While the original CSC method captured these aldehyde-containing molecules through subsequent reaction with a hydrazide-modified biotin molecule, we have found that the same selectivity for cell surface molecules can be maintained by directly capturing aldehyde-containing glycoproteins on a resin with an immobilized hydrazide group attached, a method referred to as Direct Cell Surface Capture (D-CSC) [23]. In this work, we adapt D-CSC to label luminal vessel proteins in vivo through terminal perfusion directly into the brain via the common/internal carotid. We demonstrate that this in vivo glycocapture method is highly specific for cell surface proteins and is capable of identifying the majority of known BBB carrier targets in both rat and mouse animal models. The list of identified BBB luminal proteins presented here represent a valuable resource for future selection of RMT receptors to target for BBB carriers.

## Methods

### Animals

All experiments were performed in accordance with the Canadian Council on Animal Care and were approved by the Animal Care Committee of the National Research Council Canada. Wistar rats (male, 175–200 g) and four-week male Balb/C mice were purchased from Charles River Laboratories Inc. (Montreal, Canada). Upon arrival, animals were housed in the animal facility of the National Research Council Canada for at least 5 days on a 12-h light–dark cycle at 22 ± 3 °C with free access to food and water. Details on animals used for each analysis is provided in Additional file 4: Table S1.

### Rat brain perfusion

Rats were deeply anesthetized under isofluorane gas, placed in supine position, and given a bolus i.v. injection of heparin (300 units/0.3 ml) via the right jugular vein. The left common carotid artery (CCA) was exposed and a perfusion line catheter made from polyethylene tubing (PE50) and filled with saline was inserted into the CCA and secured. Prior to perfusion both jugulars were cut, the chest cavity opened and the heart was cut. All animals were terminally-perfused via the CCA. Non-oxidized control rats (CTL) were perfused for 10 min at a rate of 2.5 ml/min with Live Cell Imaging Solution buffer (LCIS pH adjusted to 6.5; Life Science, cat# A14291DJ, containing 140 mM NaCl, 2.5 mM KCl, 1.8 mM CaCl_2_, 1.0 mM MgCl_2_ and 20 mM Hepes). Oxidized/glycocaptured rats (OXY), were perfused for 8 min (2.5 ml/min) with freshly prepared Oxidation Solution (10 mM NaIO4 dissolved in LCIS pH 6.5) followed by 2 min perfusion (2.5 ml/min) of Quench Solution (30 mM Na2SO3 dissolved in LCIS pH 6.5). At completion of perfusion, brains were dissected from the skull, weighed, and cut in half. The two hemispheres were frozen on dry ice and kept at – 80 ºC until processed for vessel isolation.

### Mouse brain perfusion

In situ brain perfusion was performed as described previously with slight modifications [24, 25]. Briefly, mice were deeply anesthetized by intraperitoneal injection of xylazine/ketamine (8/140  mg/kg). Then, the left common carotid artery was ligated at the heart side, and the external carotid artery at the bifurcation level to ensure that all the perfusate was directed straight to the brain. A polyethylene catheter filled with heparin (25  IU/mL) was then inserted in the left common carotid. The different solutions were perfused at a flow rate of 2.5 ml/min immediately after cutting the heart. The perfusion was performed in two subsequent steps: [1] eight minutes of Oxidation Solution, [2] two minutes of Quench Solution. In the control group, the first step was performed using only LCIS buffer with a pH 6.5, and the second step was performed as described above. After the perfusion, brains were extracted and the perfused hemispheres were kept at – 80 ºC until processed for vessel isolation.

### Vessel isolation and lysis

Vessels were isolated from either fresh- or snap-frozen brain tissues. Cerebellum was removed. In some cases, the perfused brain hemispheres from 2 animals were combined for vessel isolation (see Additional file 4: Table S1). Minced brains were resuspended in ice-cold PBS + 20 mM sodium sulfite with 25 µL of protease inhibitor cocktail (Sigma, Cat# P8340) prior to homogenization with a Dounce homogenizer. The homogenate was filtered through a series of pluriStrainers® (pluriSelect, San Diego, CA) stacked in descending order from 300, 100, and 20 μm pore size. The pluriStrainers were fitted into a connector ring within a 50 ml conical tube and the homogenate was transferred onto the filter stack and filtered through the strainers using gentle suction. Ice-cold PBS (5 mL; Wisent, Saint-Jean-Baptiste, QC) was used to rinse the stacked strainers, then strainers were placed upside down in a new 50-mL conical tube and rinsed with 5 ml of ice-cold PBS per strainer to collect the vessels and capillaries. To release the vessels from the strainer, the buffer was forced through the filter by pipetting up and down with a 5 mL pipette. Microvessels and capillaries collected onto 100 μm and 20 μm strainers were collected in the same 50 mL conical tube and were centrifuged at 900 × *g* for 5 min at 4 °C to pellet the vessels. The supernatant was discarded and proteins were extracted immediately from the vascular pellet by adding 1 mL (rat) or 0.5 mL (mouse) of Hydrazide Coupling Buffer (‘Hz CB’: 100 mM NaOAc, pH 5.5, 150 mM NaCl, 0.5% SDS, 1:200 v/v protease inhibitors and 1:1000 Benzonase) supplemented with 10 mM Na_2_SO_3_, and rotating the extract for 45 min at RT. Protein extracts were then frozen and stored at – 80 °C until glycocapture. Protein concentration was determined using a DC protein assay (Bio-Rad).

### BBB glycocapture and digest

The protocol for hydrazide capture was based on previously published methods [26–28] with some modifications. Mouse (0.5–0.7 mg protein) or rat (2 mg protein) vessel extracts from perfused animals were diluted to between 0.5 and 1.1 mg protein/mL with Hz CB. Samples were incubated O/N at RT with 50 µL (mouse) or 100 μL (rat) of prewashed Affi-gel hydrazide beads (Bio-Rad). Beads were washed eight times with urea buffer (8 M urea, 0.4 M NH_4_HCO_3_) and four times with 50 mM NH_4_HCO_3_, followed by reduction (10 mM DTT for 1 h at56°C) and alkylation (25 mM iodoacetamide for 1 h at RT in the dark). Beads were then washed four times with either 50 mM NH_4_HCO_3_/15% acetonitrile before incubation O/N at 37°C with 10μg of trypsin in 450 µL of the same buffer or, in rat samples only, four times with 50 mM NH_4_HCO_3_ before incubation O/N at 25 °C with 10 μg of chymotrypsin in 450 μL of 50 mM NH_4_HCO_3_. The protein yield from the mouse vessel extract was too limited to allow use of both proteases. Trypsin-released or chymotrypsin-released peptides were collected and beads were washed three times with 1.5 M NaCl, three times with 60% aceonitrile-0.1% TFA, three times with 100% MeOH, and six times with 50 mM NH_4_HCO_3_ before an incubation O/N at37 °C with 4 units (mouse) or 6 units (rat) of PNGase F (Sigma) in 100 μL of 50mM NH_4_HCO_3_. N-linked peptides were collected by combining the supernatant with further elutions with 200 μL of 50 mM NH_4_HCO_3_ and 200 μL of 50% acetonitrile/5% acetic acid. All eluates were pooled, dried under vacuum, and resuspended in 35 uL (mouse) or 50 μL (rat) of deionized water containing the glycocaptured peptide digests.

### SV-ARBEC cell surface and brain lysate glycocapture

For brain lysate glycocapture, whole brain tissue was thawed, cut into smaller pieces, and homogenized in Hz CB without SDS. After homogenization, SDS was added to a final concentration of 0.5% and samples were agitated for 1 h at RT and centrifuged at 16000 g for 10 min at RT to remove insoluble debris, and the protein concentration of the supernatant was determined using a DC protein assay (Bio-Rad). Glycocapture was performed as described for the rat vessel extracts except that proteins were oxidized in the whole brain lysate by using 15 mM Na-m-periodate for 1 h at RT in the dark, following the initial dilution into Hz CB. This reaction was then quenched by adding 30 mM Na_2_SO_4_ for 10 min at RT before proceeding with overnight RT incubation with 50 μL of prewashed hydrazide beads and following the rest of the steps outlined above. For SV-ARBEC surface glycocapture, cells were first oxidized on the plate prior to glycocapture following the previously described protocol [23].

### Mass spectrometry

Peptide digests (10–30% of each sample) were analyzed by automated nanoLC-MS(/MS) on an Eclipse Tribrid mass spectrometer coupled to a Dionex Ultimate 3000 UPLC system (Thermo Scientific). Peptides were trapped using an inline MicroTrap C18 (Phenomenex, 10 × 0.3 mm) and separated on a 10 cm × 100 μm I.D. C18 column (Waters, 1.7 μm BEH130C18, 186003546) at 500 nL/minute using a 60 min gradient (solution A: 0.1% formic acid, solution B: 100% ACN/0.1% formic acid), followed by a 9 min equilibration at 1% solution B. MS spectra were acquired in the Orbitrap between 375 and 1800 Da m/z in profile mode at 120k resolution, while data-dependent rapid CID MS/MS scans were acquired in the ion trap in centroid mode with an intensity threshold of 1.5e4, a 1.6 Da isolation window, and normalized collision energy of 35%, and a 1 s cycle time. Dynamic exclusion was enabled (60s within 10 ppm), AGC was set to “custom”, and max inject time was set to “dynamic”.

### MS data analysis

Thermo raw files were converted to mzML using MSConvertGUI Version: 3.0.18278-ad334f4d5 (parameters: peakPicking cwt snr = 0.1 msLevel = 1-). MS2 data were searched against the mouse (11Aug2022-21986 entries) or rat (12Aug2022-22860 entries) Uniprot database with decoys added using the Pyteomics Python package in mode ‘shuffle’. The database search and scoring were done using MSFragger v3.5 and Philosopher v 4.4.0 as implemented in Fragpipe v18.0 (Parameters: 20 ppm precursor tolerance, 0.2 Da fragment tolerance with mass calibration and optimal parameter search, enzyme = trypsin (2 missed cleavage) or chymotrypsin (3 missed cleavage), fixed modification: C (carbamidomethyl), variable modifications: M (oxidation), N (deamidation), write decoys selected, file output = tsv file) [29, 30]. The tsv output files from MSFragger, which included decoy database hits, were subsequently filtered using a Python script to only keep peptide-spectrum-match (psm) rows that contained at least one consensus N-glycosylation sequence (N-X(!P)-S/T/C) and a number of deamidation on N modifications that was between 1 and x inclusive, where x is the number of consensus N-glycosylation sites on the peptide. Next, the proteins represented by the remaining psm’s were filtered to keep proteins with a Protein Prophet score >  = 0.75 or > 0.8 to keep the FDR less than 1%, based on remaining decoy hits.  Supplementary data contains detailed protein identification lists for each individual experiment (Additional File 1: Data S1) and the peptide-spectrum match details (Additional File 2: Data S2).

### Data analysis

To build a list of mouse/rat cell surface proteins, we used the human cell surface list published in Sobotzki et al. [31] and converted the Gene Symbols to mouse using SynGo [32]. For proteins identified by in vivo glycocapture that were not on this published list, proteins were added to the cell surface list manually only if the Uniprot subcellular location for the mouse or equivalent human gene was listed as “cell membrane” and other Uniprot information was consistent with extracellular plasma membrane expression. Functional classification and Gene Ontology (GO) enrichment analysis were implemented in Panther v 17.0 [33, 34] using Panther Go-Slim categories and Reactome pathway with the Binomial Test type and FDR correction for the statistical overrepresentation test. Single-cell RNAseq data was downloaded from the Allen Mouse Brain Atlas. http://portal.brain-map.org/atlases-and-data/rnaseq/mouse-whole-cortex-and-hippocampus-10x; file = “Gene Expression by Cluster, median (.csv))” [35]. To simplify the analysis, the number of cell clusters were minimized by combining all consecutive cell clusters with the same description name after the cell number keeping only the highest expression value for each gene. This simplification reduced the cell type clusters from 388 to 75. Outlier analysis of cell clusters were identified using ROUT (Q = 0.1%) implemented in Prism 9.5.1.

## Results

### *Identification of luminal BBB proteins in rats by *in vivo* glycocapture*

The workflow developed for the in vivo glycocapture of proteins present on the blood-accessible surface of the BBB is described in Fig. 1. In brief, rodents were anesthetized and the brain perfused with a mild oxidizing solution to remove blood and to convert the cis-diols present in glycans to aldehydes, a reactive group that is rarely present in naturally occurring proteins (Fig. 1B). Animals in the control group were perfused only with buffer that did not contain any oxidation reagent. Brains were then extracted and homogenized on ice prior to enrichment of microvessels and capillaries via a filtration-based method [36–38]. The enriched microvessel fraction was lysed and solubilized in an SDS detergent-containing buffer to ensure that all membrane proteins were fully solubilized. To isolate the proteins located on the luminal side of the brain microvasculature, the aldehyde-containing oxidized glycoproteins were covalently captured onto beads modified with a hydrazide group and extensively washed (Fig. 1C). Next, the immobilized glycoproteins were digested with a protease, either trypsin or chymotrypsin, releasing non-glycosylated peptides from the immobilized glycoproteins (“protease-released fraction”) and leaving only the glycosylated peptides attached to the beads. Lastly, PNGaseF, an enzyme that cleaves the bond between an N-linked glycan and asparagine, is used to release N-glycosylated peptides (“N-glycosylated fraction”).

Altogether, we collected samples from 10 individual rats using the above workflow, half of which were perfused with the mild oxidation solution (‘OXY’) and half perfused with buffer (‘CTL’), in order to confirm the specificity of the labelling approach. In theory, glycoproteins should only be labelled and captured after oxidation, so any proteins that are identified with the CTL perfusion are likely proteins with naturally-occurring aldhehyde modifications or proteins that bind to the capture beads due to non-specific interactions. As can be seen in Additional file 3: Figure S1A (MSight), peptide signals from the N-glycosylated fraction are visible in both the CTL and OXY samples when the mass spectrometry data is visualized in a 2D representation using MSight [39]. As would be expected, there are significantly more peptide spots present in the OXY samples; these represent glycopeptides that have been specifically enriched through our in vivo glycocapture method. In contrast, the protease-released fraction showed similar peptide patterns between CTL and OXY (Additional file 3: Figure S1B) and thus was not analyzed further. In all cases, the peptide spot pattern is quite similar between individual animals receiving the same treatment showing reproducibility in the approach.

**Fig. 2:**
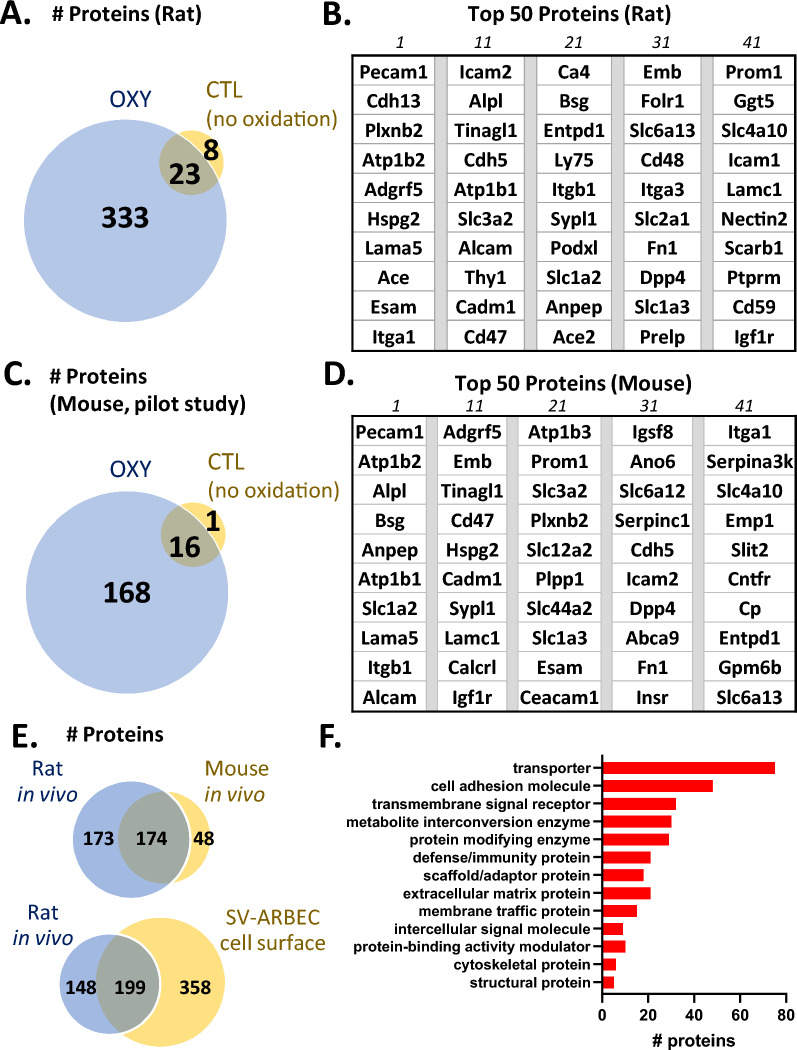
In vivo glycocapture enriches for BBB proteins with high specificity in both rat and mouse. **A** Number of proteins identified in rats perfused with oxidation solution (OXY) or a buffer control (CTL) after the glycocapture workflow. **B** Top 50 proteins ranked by spectral counts that were identified in OXY rat samples. All proteins are listed by their corresponding HGNC gene symbol. **C** Number of proteins identified in a pilot mouse study perfused with oxidation solution (OXY) or a buffer control (CTL). **D** Top 50 proteins ranked by spectral counts identified in all OXY perfused mice. **E** Comparison of proteins identified by in vivo glycocapture in rat and in mouse (top) and comparison of proteins identified by in vivo glycocapture in rat with proteins identified by cell surface capture of the rat endothelial cell line, SV-ARBEC (bottom). **F** Classification of all proteins identified by in *vivo* glycocapture in either rat or mouse by Panther protein class

Using typical proteomic database search methods, the peptides present in the LC–MS data of the N-glycosylated fraction were identified and assigned to proteins to identify proteins present at the BBB. Following rigorous filtering of the rat data to maximize identifications while minimizing false positive identifications below 1% (see Methods), 356 proteins were identified in the OXY samples while only 31 proteins were identified in the CTL samples. Figure 2A shows that 23 of the 31 proteins identified in the CTL sample were also found in the OXY samples; of these 23 proteins, 14 were found at higher levels in the OXY fraction relative to the CTL fraction, with 1.5–64-fold more spectral counts assigned to them in the OXY fraction. Based on this observation, we suspect that some of the CTL hits may represent carry-over in the nano-LC columns. In contrast, the other 9 proteins identified in both the CTL and OXY fractions had similar spectral counts in each sample, and likely represent background binding. These 9 proteins include common abundant proteins like histones and tubulins, together with a few predicted surface receptors (Lrp1, Atp1a3, and Slc6a11) which possibly contain naturally occurring aldehyde modifications, such as carbonylation. These 9 proteins were eliminated from our final list of rat proteins identified at the luminal surface of the rat BBB, resulting in the identification of 347 rat proteins (Additional file 4: Table S2). The 50 proteins with the highest number of spectral counts are highlighted in Fig. 2B.

### *Identification of luminal BBB proteins in mice by *in vivo* glycocapture*

A more complete understanding of the differences in protein composition at the BBB between species would be useful for the selection of animal models for various types of BBB studies. For this reason, we decided to extend our study into mouse, another common BBB model species. In some cases, two perfused hemispheres from different animals were combined prior to vessel enrichment (see Additional file 4: Table S1). Due to the smaller size of mouse brains, which resulted in a lower amount of total protein present in the enriched vessel lysate prior to glycocapture, we used only a single protease (trypsin) to avoid splitting this small sample into two separate digests. First, a pilot study using 8 mice was performed to confirm that the specificity of glycocapture was similar to the rat experiment. In this experiment vessels were pooled from two animals for each condition (CTL or OXY) prior to glycocapture, resulting in two CTL samples and two OXY samples in the LC–MS analysis. In this pilot study, 184 proteins were identified in the OXY samples while 17 proteins were identified in the CTL samples (Fig. 2C). Of the proteins identified in the CTL, 16 were also found in the OXY fraction and 12 of these proteins had higher spectral counts in the OXY sample (1.5–18-fold more than CTL samples), suggesting enrichment by oxidation labelling. To increase the number of proteins identified at the mouse BBB, we perfused an additional five mice with oxidation solution and compiled the proteins identified from all OXY perfused animals, removing the 4 proteins that were identified at equal levels in the CTL and OXY samples during our pilot study. This analysis led to the identification of 224 proteins present at the luminal BBB surface in mice (Additional file 4: Table S3). The 50 proteins with the highest number of spectral counts in mice are highlighted in Fig. 2D.

There is strong overlap between the proteins identified at the BBB in rat and in mouse (Fig. 2E, top panel). A combined list of proteins identified in either species contains 395 proteins, with 174 proteins found in both species (Additional file 4: Table S4). A large number of proteins (173) were identified only in rat and not in mouse, likely due to technical reasons, including the lower amount in input protein in mouse due to the smaller brain size and our subsequent decision to use only one protease (trypsin) instead of two (trypsin and chymotrypsin). A high proportion of the proteins identified by in vivo glycocapture in rat and mouse vessels are cell surface proteins, with 90% of the proteins predicted as cell surface and an additional 6% of protein predicted as secreted or extracellular matrix proteins that are often associated with the cell surface. These proteins contain a large number of proteins classified as transporters, cell adhesion molecules, and transmembrane signal receptors, as would be expected at the BBB (Fig. 2F) and contains several acknowledged endothelial cell markers, including PECAM1, VWF, CD34, Cldn5, ZO1 (Tjp1), and the majority of known BBB carriers, including insulin receptor (Insr), transferrin receptor (Tfrc), and Insulin-growth factor 1 receptor (Igf1r). The low number of proteins classified as cytoskeletal proteins highlights the specificity of our labelling and analysis method.

To obtain an initial indication of the similarity between our in vivo glycocapture results and proteins expressed on endothelial cells, we compared the proteins identified by rat in vivo glycocapture with those found on the cell surface of an immortalized adult rat brain microvascular endothelial cell line, SV-ARBEC (Fig. 2E, bottom panel) [40]. In total, 557 proteins were identified on the surface of SV-ARBEC cells by surface glycocapture using similar labelling and analysis conditions as those used in the in vivo glycocapture, as listed in Additional file 4: Table S5. Of these, 199 proteins were also found in the rat in vivo glycocapture; therefore over 57% of the proteins identified by in vivo glycocapture were also found in endothelial SV-ARBEC cells.

### *Comparison of *in vivo* glycocapture to brain lysate glycocapture*

**Fig. 3:**
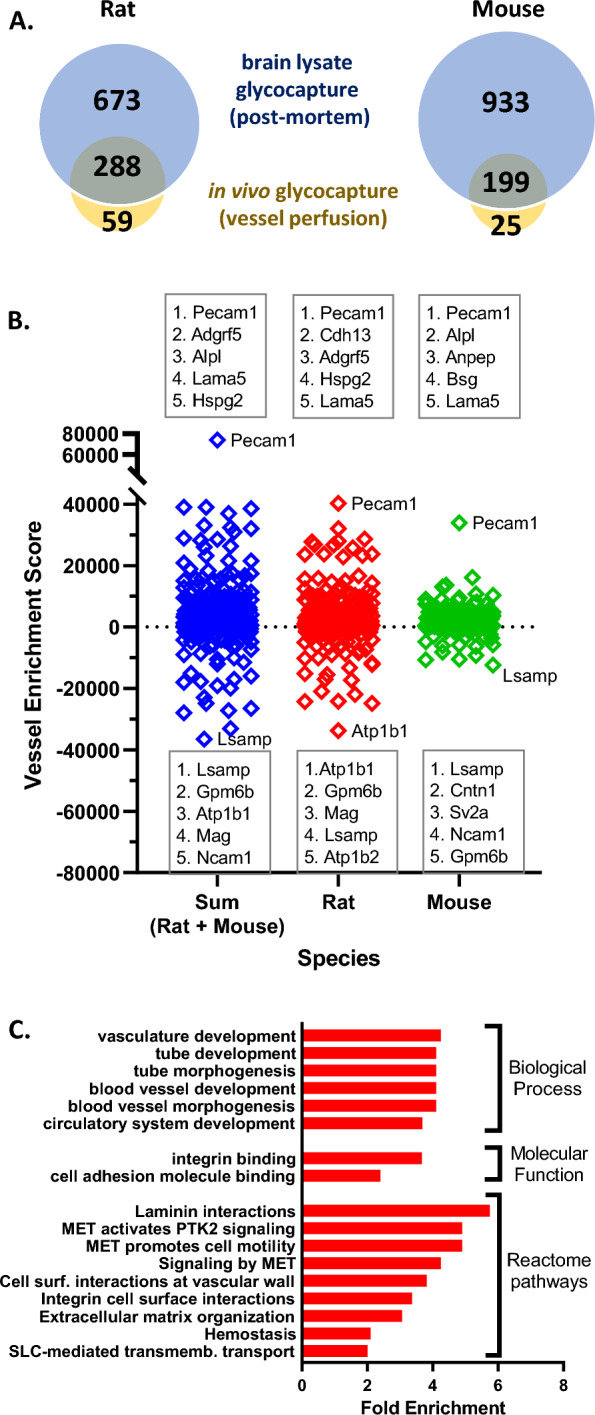
Comparison of in vivo glycocapture to a whole brain lysate glycocapture. **A** Number of proteins identified by in vivo glycocapture and by brain lysate glycocapture in rat and mouse. **B** Distribution of the vessel enrichment scores for all proteins identified proteins by in vivo glycocapture with the highest scoring and lowest scoring proteins indicated. **C** Gene ontology and Reactome pathway enrichment analysis for the 194 in vivo glycocapture proteins with a vessel enrichment score > 1000. All listed categories had at least a twofold enrichment value with an FDR p-value < 0.05 when using a reference of the 1367 proteins identified in all glycocapture experiments, including both in vivo glycocapture and brain lysate glycocapture

To provide an indication of the proteins identified when glycocapture labelling is not restricted to the perfused vessels, we also performed glycocapture on a complete brain lysate by oxidizing all glycoproteins present within a solubilized brain lysate in both rat and mouse. More proteins were identified in the brain lysate glycocapture than by in vivo glycocapture (Fig. 3A), likely due to the less restricted labeling procedure, and the higher amount of starting material for mouse (2 mg vs. 0.5–0.7 mg for the in vivo glycocapture; 2 mg input was used in both cases for rat). In rat, 961 proteins were identified in the brain lysate glycocapture, compared to the 347 that were identified in the in vivo glycocapture. In mouse, this difference was even larger since the whole brain input was much less restricted by brain size than the in vivo glycocapture, resulting in 1132 and 224 proteins identified in whole brain and in vivo glycocapture respectively. The overlap in identifications between brain lysate glycocapture and in vivo glycocapture is high as highlighted in Fig. 3A. All identifications from the rat and mouse brain lysate glycocapture are listed in Additional file 4: Table S6.

To identify proteins that are enriched in vessels, relative to the whole brain, we calculated a “Vessel Enrichment score” (VE score) for each identified protein. First, spectral counts associated with identified proteins in each dataset were converted to counts-per-million (CPM) to normalize between datasets. The VE score was then calculated for each protein by subtracting the CPM for that protein in the brain lysate glycocapture from the CPM in the in vivo glycocapture dataset. Thus, a positive VE score reflects a higher relative abundance for that protein in the vessel dataset relative to the whole brain dataset. The majority of the proteins in the in vivo glycocapture dataset show a positive VE score (71% rat, 83% mouse, 76% combined species). To calculate VE scores for combined species data, the VE scores from each species were normalized to the median VE score for that species and then summed. The distribution of VE scores in the mouse and rat data, as well as the combined data set are shown in Fig. 3B with the 5 proteins with the highest and lowest VE scores in each dataset highlighted. Many of the proteins with the highest VE scores are well-known vessel marker proteins [41], such as Pecam1 (CD31), Anpep (CD13), Bsg (CD147), and Alpl (Tissue nonspecific alkaline phosphase; TNAP), with Pecam1 having the highest score in all samples. In contrast, proteins with the lowest VE scores contain many proteins known to be widely expressed in neuronal cells, such as Lsamp, Ncam1, Mag, and Sv2a.

Next, we characterized the proteins identified with a high VE score (> 1000) using a statistical overrepresentation test implemented by Panther [33]. As can be seen in Fig. 3C, several Gene Ontology (GO) terms were enriched with FDR-corrected p-values < 0.05, including several GO terms associated with vasculature and blood vessel development, as well as integrin and cell adhesion molecule binding. A number of Reactome pathways were also enriched in this set of proteins, including several MET signaling pathways, cell surface interactions at the vascular wall, and SLC-mediated transmembrane transport. Taken together this enrichment analysis serves to further demonstrate the specificity of the proteins identified by in vivo glycocapture.

### Comparison of proteomic data to single-cell RNAseq data for cell-type analysis

**Fig. 4:**
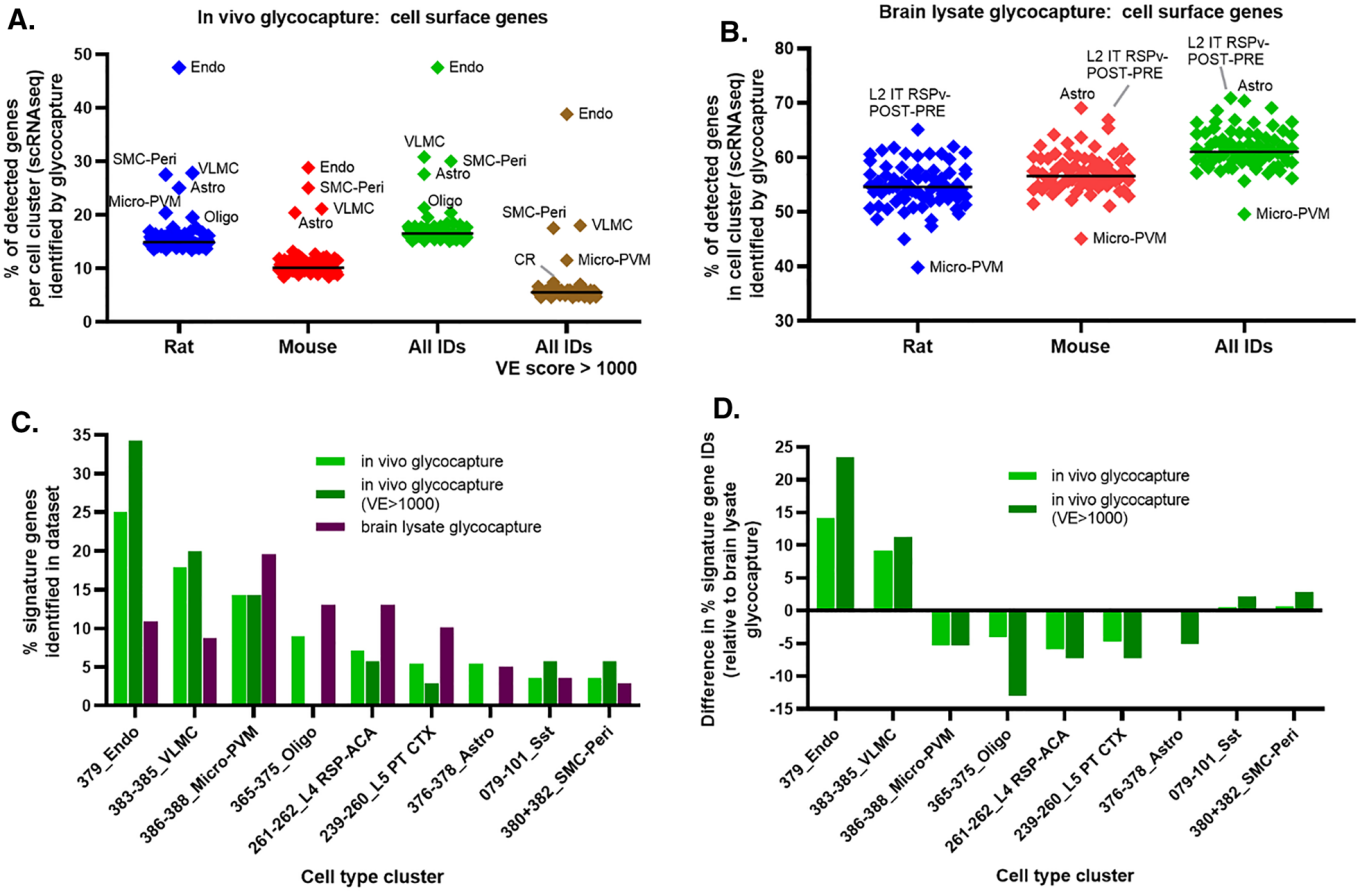
Cell-type analysis of proteins identified by in vivo glycocapture though comparison to single-cell RNAseq data. **A** For each in vivo glycocapture dataset, the identified proteins were aligned to the genes that were detected by single-cell RNAseq in individual cell clusters using data from the Allen Brain Atlas. The percent of genes detected by scRNAseq (with a read count > 0) in each cell count that were identified in the glycocapture dataset are shown. Statistical analysis comparing all cell types was performed to identify cell types that are outliers. These outlier cell types are labelled and include endothelial cells (Endo), smooth muscle cells-pericytes (SMC-Peri), vascular lepotomeningeal cells (VLMC), micro-perivascular macrophages (Micro-PVM), and astrocytes (Astro), oligodendrocytes (Oligo), and Cajal-Retzius cells (CR). **B** The same analysis described in panel A was repeated using the brain lysate glycocapture datasets. None of the cell types were tagged as outliers. A few cell types showing trends towards over or under representation are labelled. **C** The percentage of identified signature genes associated with each cell type in the listed proteomic dataset: in vivo glycocapture (light green bar), in vivo glycocapture with a vessel enrichment score > 1000 (dark green bar), or brain lysate glycocapture (brown bar). Combined species data was used for the analysis and only cell types that had at least 5% identified signature genes in at least one dataset were included in the graph. **D** The difference in percent identified signature genes between the in vivo glycocapture dataset and the brain lysate glycocapture dataset for all identified in vivo glycocapture proteins and identified in vivo glycocapture proteins with a vessel enrichment score > 1000, for the cell types listed in panel C

To explore the cell types that were most likely to be labelled using our perfusion-based labelling method, we compared the proteins identified by in vivo glycocapture to the clustered single-cell RNAseq (scRNAseq) data from mouse brain cells compiled by the Allen Brain Map project [35]. For each of the major brain cell type clusters, we calculated the percentage of cell surface genes detected by scRNAseq that were also detected by in vivo glycocapture (Fig. 4A). The majority of the 75 major cell type clusters had a very similar percentage of detected genes identified within the in vivo glycocapture dataset, as shown by the tight clustering of most cell types around the median value line. Only 4 cell-type clusters were found to be outliers in all in vivo glycocapture datasets using ROUT (Q = 0.1%) with all four of these cell-type clusters showing a higher percentage of detected genes in the in vivo glycocapture datasets than would be expected based on the data from the other cell types. Endothelial cells (Endo) contained the highest percentage of RNAseq-detected genes found at the luminal BBB with up to 48% of the detected cell surface proteins found in the in vivo glycocapture dataset. The other three cell types that were outliers in all data sets are also known to be associated with vessels. These include vascular lepotomeningeal cells (VLMC), smooth muscle cells-pericytes (SMC-Peri), and micro-perivascular macrophages (Micro-PVM). A solid level of enrichment was seen for astrocytes (Astro) in both rat and mice, but interestingly astrocytes do not appear to show a high percentage of enriched genes when looking only at BBB proteins with a VE score > 1000, likely because these cells have a high proportion of glycoproteins even in the brain lysate glycocapture since astrocytes are abundant in the brain (Fig. 4B). A very low level of enrichment in detection was also seen for oligodendrocytes (Oligo) and Cajal-Retzius cells (CR) in some datasets (see Fig. 4A). In contrast, no cells were found to be statistical outliers when the same analysis was completed with the brain lysate glycocapture dataset (Fig. 4B) though a few cell types showed a trend toward increased or decreased identifications.

Next, we analyzed “signature genes” that were detected by single-cell RNAseq in ONLY one cell type cluster. Thus, these signature genes are more likely to be specific markers of that particular cell type. This analysis complements the cell-type analysis discussed above since it does not rely on normalization to the depth of scRNAseq coverage for each cell type, which was a concern because endothelial cells and several of the others cell clusters described above show a relatively poor depth of coverage in the scRNAseq dataset, possibly due to the relative rarity of these cells in the brain. Specifically, we asked what percentage of signature genes identified in each glycocapture dataset was associated with a particular cell type cluster. Again, the highest proportion of signature genes representing endothelial cells was found in the in vivo glycocapture dataset (25%), with an even higher proportion of endothelial signature genes identified in the subset of proteins that have a VE score > 1000 (34%; Fig. 4C). A significant portion of signature genes associated with VLMC (18%), Micro-PVM (14%), and Oligo (9%) cell clusters were also identified. Interestingly, while Endo, VLMC, Sst (GABAergic neurons), and SMC-Peri cell signatures were more likely to be represented in the proteins with a VE score > 1000, cell signature genes associated with Oligo and Astro clusters were completely absent in the high VE score subset. This likely reflects the high proportion of Oligo and Astro proteins represented in the brain lysate glycocapture dataset, which was used in the VE score calculation. As a comparison, we also calculated the same values for the brain lysate glycocapture dataset, shown in the brown column. Intriguingly, only Endo and VLMC cell clusters showed more than 5% of associated signature genes in the in vivo dataset than in the whole brain dataset, as demonstrated by their positive values after correction based on the brain lysate glycocapture identifications (Fig. 4D).

## Discussion

Here we describe a novel labelling and purification workflow to identify vessel-associated proteins at the luminal BBB surface, relying on terminal perfusion of a mild oxidation reagent to oxidize the glycans of glycoproteins at the luminal surface of vessels. This approach is based on our previously published D-CSC modification of the CSC cell surface protein method [22, 23]. Our modifications removed the necessity of a separate biotinylation reaction. The simplicity of the labeling in D-CSC, which requires only a single labelling step, lends itself well to labelling luminal vessel proteins in vivo through a perfusion approach. Previous work aimed at labelling proteins at the BBB through perfusion-based methods have been recently reviewed [11] and have generally relied on the direct biotinylation of proteins through reaction of an NHS-ester with lysine residues or the N-terminus. The results with this NHS-ester based labelling method are reported to be much poorer in brain than in other organs [16, 18], perhaps reflecting challenges in the accessibility of protein molecules due to the thicker glycocalyx. The in vivo glycocapture approach described here demonstrated that direct labelling of glycans via oxidation allowed the identification of a large number of proteins while establishing a high level of specificity that has not been demonstrated with other workflows. Aldehyde-stability is a minor concern in this approach; reaction between the aldehyde on the oxidized glycan with other components in the lysate may result in loss of some labels. However, using in vivo glycocapture, we identified more than twofold the number of proteins at the BBB than have previously been identified by other perfusion-based labelling methods based on NHS-ester proteins, while also identifying a much higher percentage of cell surface proteins [11, 16–18]. The perfusion methods used here may also have played a role in our results. We utilized intracarotid perfusion for rats and mice, both of which likely result in improved perfusion of the brain relative to the intracardiac perfusion that was used in the biotinylation studies. However, preliminary studies on the in vivo glycocapture using intracardiac whole body perfusion showed similar results for brain as the ones found in this study (data not shown), suggesting that the labelling method may play a larger role in these improved results than the perfusion method does.

One advantage of the in vivo glycocapture approach, as well as the Cell Surface Capture method that it is based on, is that the specificity can be improved by filtering the peptide identifications to keep only peptides that contain a deamidated N-linked sequon as would be expected in a PNGaseF-released peptide. This extra filtering step eliminates many of the peptides that are visible in the CTL sample as well as many of the decoy hits, greatly improving the number of proteins identified while still effectively controlling the false-discovery rate (FDR). FDR control is especially challenging when spectral signal intensity is low, as it is in these experiments due to the small quantity of peptides isolated through this procedure. When the N-linked sequon filter was not used, the number of proteins successfully identified at a 1% FDR was considerably lower, mostly due to an increase in the decoy hits due to the large number of poor-quality MS2 spectra in this very low-level sample.

Many proven receptor-mediated transcytosis targets for BBB carriers were identified in this study, including insulin receptor (Insr) [42], Insulin-like growth factor receptor (Igf1r) [25], transferrin receptor (Tfrc) [43, 44], Glucose transporter type 1 (Slc2a1) [45], Low-density lipoprotein receptor-related protein 1 (Lrp1) [46], Cell cycle control protein 50A (Tmem30a) [7], 4F2 cell-surface antigen heavy chain (Slc3a2) [45, 47], Neonatal Fc receptor (Fcgrt) [48], and basigin (Bsg) [45, 49]. In contrast, a few known vessel proteins are notably absent from this list, including several of the efflux transporters, such as p-glycoprotein/MDR1 (Abcb1 in rat, Abcb1a/b in mouse) and Multidrug resistance-associated protein 2/MRP2 (Abcc2). There are several technical reasons why proteins might not be identified by in vivo glycocapture. First, there is a limit to our ability to detect proteins expressed at low-levels on the cell surface, which highly biases our results to proteins that are present at reasonable abundance. This is likely the reason why many efflux transporters were not identified—of the 4 ABC transporters identified in this study (Abcc1, Abca9, Abcg2, and Abca8a), all were found with low spectral counts in only one of the two species–suggesting that they were near the limit of detection. In many ways, this bias towards identification of abundant proteins could be considered a positive for the development of RMT carriers for BBB crossing where an abundant receptor may be preferred. To this end, it is notable that most of the well-known RMT carriers are in the top 100 proteins as ranked by vessel enrichment score, which is highly weighted by abundance at the BBB. The second major reason why proteins may not be identified by in vivo glycocapture is the absence of an N-linked glycosylation site that is located in a peptide of suitable size to be identified by the MS methods being used. In vivo glycocapture will not identify proteins that are not glycosylated, or that only have o-linked glycosylation. In early studies of in vivo glycocapture, we attempted to improve our coverage of proteins by analyzing the peptides that are released from the hydrazide beads by trypsin or chymotrypsin in the protease-released fraction (see Fig. 1). A much larger number of proteins were identified in the protease-released fraction and these identifications were somewhat enriched in cell surface proteins. However, we also found a considerable number of proteins in this fraction from the CTL animals that were perfused only with buffer (see Additional file 3: Figure S1B), suggesting that the specificity for BBB proteins was not sufficient to lead to confident identification of BBB proteins. Based on this result, we decided not to analyze this fraction further. Because of these technical limitations, a negative result (non-identification) in this in vivo glycocapture study is not necessarily indicative of the absence of a protein at the BBB.

Another major technical limitation of any perfusion-based labelling method is the difficulty in preventing transfer of the label through the vessel and into the surrounding tissue or cells. We specifically chose to use a perfusion rate that mimicked the pressure of normal blood flow to minimize any disruption of the BBB integrity and to keep microvessels completely intact. However, there is always the possibility that some labelling agent was able to leak outside the vessel at low levels. It is also possible that some of the oxidizing agent diffused into the brain through an active or passive process. We identified a number of proteins associated with the basement membrane (several laminins, nidogen 2, agrin), suggesting the possibility that the oxidation reagent may have penetrated somewhat deeper than just the luminal endothelial cell surface. However, it remains possible that these extracellular matrix proteins are present in lower amounts on the luminal cell surface. The cell-type analysis highlighted the possibility that some non-endothelial cells were also labelled by our method. While the highest enrichment was seen for genes that are known to be expressed in endothelial cells themselves, we also saw some enrichment in genes associated with SMC-Peri, VLMC, Micro-PVM, Astro, and Oligo cells suggesting the possibility that some of these cells may also be labelled in our method. Alternatively, the apparent enrichment in proteins associated with these non-endothelial cells may also be due to an artifact caused by the inherent similarity in gene expression between these other cell types and endothelial cells, coupled with the stochastic, random nature of detection of lower-level genes in scRNAseq data. In fact, the cell types that showed the strongest enrichment in our analysis (SMC-Peri, VLMC cells) loosely co-cluster with endothelial cells in the Allen brain map data [35]. Thus, it remains possible that only endothelial cells were labelled in our experiment and that these other cell types were only highlighted due to their similar expression to endothelial cells. The relative low depth of coverage of these rarer cell types, as evidenced by the smaller number of genes detected, may also affect this analysis.

Similar to proteomic data, scRNAseq data also has an underrepresentation of cell surface proteins due to their relatively low expression levels compared to intracellular proteins involved in metabolism or the cytoskeleton. This leads to a higher percentage of non-detection events for cell surface proteins than more abundant protein types in scRNAseq data, which is one reason why we focused on cell surface genes/proteins in our comparative analysis. In fact, while 11.6% of all genes encode cell surface proteins, only 7.1% ± 1.0% of the genes detected by scRNAseq in each of the cell type clusters encode cell surface proteins (data not shown). In an outlier analysis, a larger percentage of cell surface protein-encoding genes were identified only in astrocytes, where 12.4% of detected genes encoded cell surface proteins, which likely is the cause of the higher percentage of brain lysate glycocapture proteins represented in astrocytes relative to other cell types in Fig. 4A, B.

Many cell surface proteins were identified by in vivo glycocapture. For further study as potential RMT carrier targets, the best options are likely to be proteins that were identified in both mouse and rat with high VE scores. These would include proteins such as Anpep, Esam, Icam2, and Alpl, several of which are known to play a role in endocytic processes including leukocyte extravasation [50–53]. However, it is important to recognize that the VE score is highly biased towards more abundant BBB proteins that contain larger extracellular domains and more accessible N-linked glycopeptides that happen to be well-suited to mass spectrometry analysis. Therefore, not all proteins with lower VE scores should be discounted and any protein with a positive VE score may be considered as a possible RMT candidate. For instance, one of the best-studied and most highly validated RMT targets, transferrin receptor (Tfrc) [54–56], was identified in this study with a VE score of 3617 (83rd percentile; rank 55 out of 347) in rat, but with a much lower VE score of 227 (20th percentile, rank 179 out of 224) in mouse. In this case, the apparent difference in VE scores between the two species appears to be due to technical reasons. Upon manual inspection of the peptide-spectrum-match data, two different unique N-glycopeptides were identified from Tfrc in rat, while only one unique N-glycopeptide was identified in mouse. Furthermore, this one N-glycopeptide had a very late elution time, which makes it more likely for this peptide to be “lost” due to minor variability in the nano-LC column performance between runs. In fact, a closer inspection of the data suggests this may be the case; even in the rat data, one particular MS run showed a much higher identification of this Tfrc peptide relative to other runs. For this reason, the VE score should be considered as a useful tool for prioritizing possible RMT targets; however, it is also important to evaluate other criteria and additional datasets, as has been previously discussed in the literature [45, 57].

Another example of how technical details can affect the results is Cd59, an interesting potential novel BBB carrier candidate that was found in rat with a high enrichment score, but was not identified at all in mouse. Cd59 is known to be expressed in endothelial cells [58, 59], including in brain as shown by tissue IHC in Protein Atlas (https://www.proteinatlas.org/) [60]. In addition, Cd59 was calculated to be 68% luminal and 32% abluminal in HCBEC/D3 cells based on apical and basolateral membrane fractionation by sucrose gradient [61] (data not shown). In the in vivo glycocapture experiments, the observed species difference is likely due to differences in the sequence between mouse and human Cd59. Cd59 derives from only one gene in rat, while mouse contains two highly similar Cd59 proteins, Cd59a and Cd59b, with Cd59b showing restricted expression in testes while Cd59a is expressed more widely, including in endothelial cells, similar to Cd59 in rat and human [62]. All Cd59 proteins have only one N-linked glycosylation site. While Cd59b has an N-linked peptide similar to rat Cd59, the N-linked glycosylation site in Cd59a is present in an N-linked tryptic peptide that is too long to be easily identified by LC–MS under the conditions used in these experiments, explaining why this protein was not identified at the mouse BBB by in vivo glycocapture.

A few proteins predicted to be present on the membranes of intracellular organelles or vesicles were identified in these experiments, with Synaptophysin-like 1 (Sypl1) and the cation-dependent mannose-6-receptor (M6pr, also known as MPR46) showing the highest vessel enrichment score. The identification of these protein by in vivo glycocapture with a relatively high vessel-enrichment score suggests that they may be expressed on the endothelial cell surface, or possibly in extracellular vesicles that are present at the BBB interface. However, it also remains possible that these proteins were identified due to local leakage of the labelling agent from the vessels or due to labelling of another cell-type interacting with the BBB (such as T cells) which may have been labelled through perfusion. It is intriguing that M6P-mediated uptake at the BBB has been shown to be very low in adult mice [63, 64], although we only identified M6pr in rat, not in mouse, so the possibility of a species difference remains. Interestingly, the cation-independent mannose-6-phosphate receptor (Igf2r, also known as MPR300 and MPRI), was also identified in rats by in vivo glycocapture and there is some evidence that this protein may be present in brain capillary endothelium in cows and pigs [65]. Lastly, it remains possible that M6pr could be present in mouse, but is held in an inactive state at the BBB. Additional experiments would be required to distinguish from these possibilities.

In addition to the integral cell membrane proteins that were identified in these experiments, a number of secreted proteins were identified as well. The highest scoring secreted proteins by vessel enrichment score included Tubulointerstitial nephritis antigen-like 1 (Tinagl1) and transferrin (Tf). These secreted proteins were likely labelled at the BBB due to their tight interaction with cell receptors that are present on endothelial cells, including integrins α5β1 and αvβ1 for Tinagl1 [66] or transferrin receptor (Tfrc) for Tf, all of which were also identified by in vivo glycocapture.

In this work, a few proteins that would not be expected on the luminal endothelial cell surface were identified, such as Slc1a2 and Slc1a3, which are thought to be mainly expressed on astrocyte endfeet and are therefore associated with the abluminal surface [67]. Interestingly, Slc1a3 was identified in our rat SV-ARBEC cell glycocapture study showing that Slc1a3 is present in endothelial cells, at least in culture. As discussed above, it is possible that the mild oxidation reagent was able to penetrate the endothelial cell and label some abluminal proteins. Alternatively, this protein may be found at some level on the luminal surface, likely at a lower level than on the abluminal surface. Unfortunately, confirming luminal expression of the proteins identified in this work using orthogonal methods is extremely difficult. The luminal and abluminal membranes are separated by only 300–500 nm in human brain microvessels, making these two membranes difficult to resolve by typical light microscopy immunohistochemistry approaches [68, 69]. Electron microscopy studies can distinguish the two endothelial surfaces with high resolution, but require care in selecting the antibody and fixation conditions which can affect relative labelling of luminal and abluminal surfaces [70] while also requiring specialized expertise and significant time to scan vessels throughout the entire brain to identify potential differences in polarization in different brain areas or capillary structures. Interestingly, but perhaps not surprisingly, different endothelial cells have been shown to have different polarization of some transporters such as GLUT1 [68], suggesting that the same may be true for other BBB proteins, further complicating the issue. In a complementary approach, luminal and abluminal membranes can be isolated by fractionation [71, 72], but this appears to be an enrichment rather than a pure separation making interpretation somewhat difficult and requiring correction for contamination [72]. Additional methods to determine the abluminal and luminal expression of proteins at the BBB is full of challenges and remains an area worthy of further method development.

### Next steps

In this work, we have demonstrated the use of in vivo glycocapture to profile the proteins present at the blood–brain barrier in rat and mouse. In future work, we hope to implement relative quantification of these identified proteins to evaluate how protein expression changes at the BBB in various disease models, including Alzheimer’s and cancer. The application of in vivo glycocapture could also be expanded beyond the brain by using whole body perfusion to identify vessel proteins present in peripheral organs such as kidney, lung, and liver. Lastly, extension of this work into other species, including non-human primates, would be of great benefit to the field.

## Conclusions

We have developed a novel perfusion-based labelling method that enabled the specific isolation and identification of proteins exposed to the luminal surface of microvessels in the brain of rats and mice. The majority of proteins identified were cell surface proteins and many known BBB receptors and transporters were found. The resulting list of identified proteins will serve as a valuable resource for future BBB studies.

### Supplementary Information

**Additional file 1: Data S1.** Individual protein identification lists for all datasets.

**Additional file 2: Data S2.** MSFragger peptide-spectrum match (psm) details after filtering for all datasets. Note that this table includes hits to the decoy database (if applicable).

**Additional file 3: Figure S1.** Example LC-MS data for control and oxidized rats.

**Additional file 4: Table S1.** Sample and MS run information. **Table S2.** Proteins identified by in vivo glycocapture from rat brain. **Table S3.** Proteins identified by in vivo glycocapture from mouse brain. **Table S4.** All proteins identified by in vivo glycocapture from rat or mouse brain. **Table S5.** All proteins identified by cell surface glycocapture in a rat endothelial cell line (SV-ARBEC). **Table S6.** All proteins identified by cell surface glycocapture in a rat endothelial cell line (SV-ARBEC).

## Data Availability

The full peptide identification results generated during the current study are available as Supplementary Data within this manuscript. All materials are available from commercial sources.

## Abbreviations

BBB: Blood–brain barrier
CCA: Common carotid artery
CPM: Counts per million
CSC: Cell surface capture method
CTL: Control/buffer-perfused animals
D-CSC: Direct cell surface capture method
GO: Gene ontology
Hz CB: Hydrazide coupling buffer
LC–MS: Liquid-chromatography coupled mass spectrometry
MS: Mass spectrometry
NHS: N-hydroxysuccinimide
NVU: Neurovascular unit
OXY: Oxidized/oxidation reagent perfused animals
RMT: Receptor-mediated transcytosis
scRNAseq: Single-cell RNAseq
VE: Vessel enrichment
