# Supplementary material for: The proteome of the blood–brain barrier in rat and mouse: highly specific identification of proteins on the luminal surface of brain microvessels by in vivo glycocapture
Source: Fluids Barriers CNS. 2024 Mar 4;21:23. doi: 10.1186/s12987-024-00523-x (PMC10910681; doi:10.1186/s12987-024-00523-x)
Supplement: Supplementary file 3 — Additional file 3: Figure S1. Example LC-MS data for control and oxidized rats. [file 12987_2024_523_MOESM3_ESM.pdf]

A.

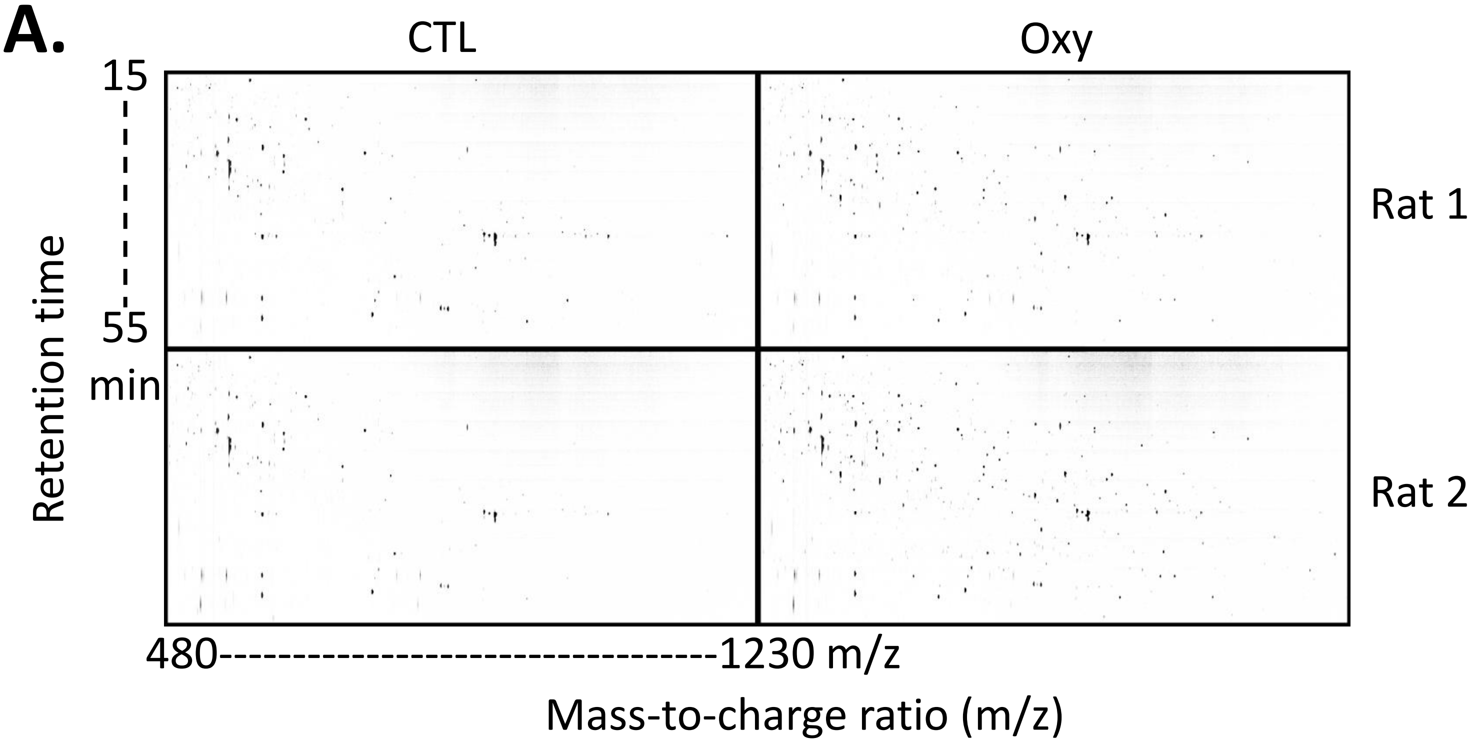

B.

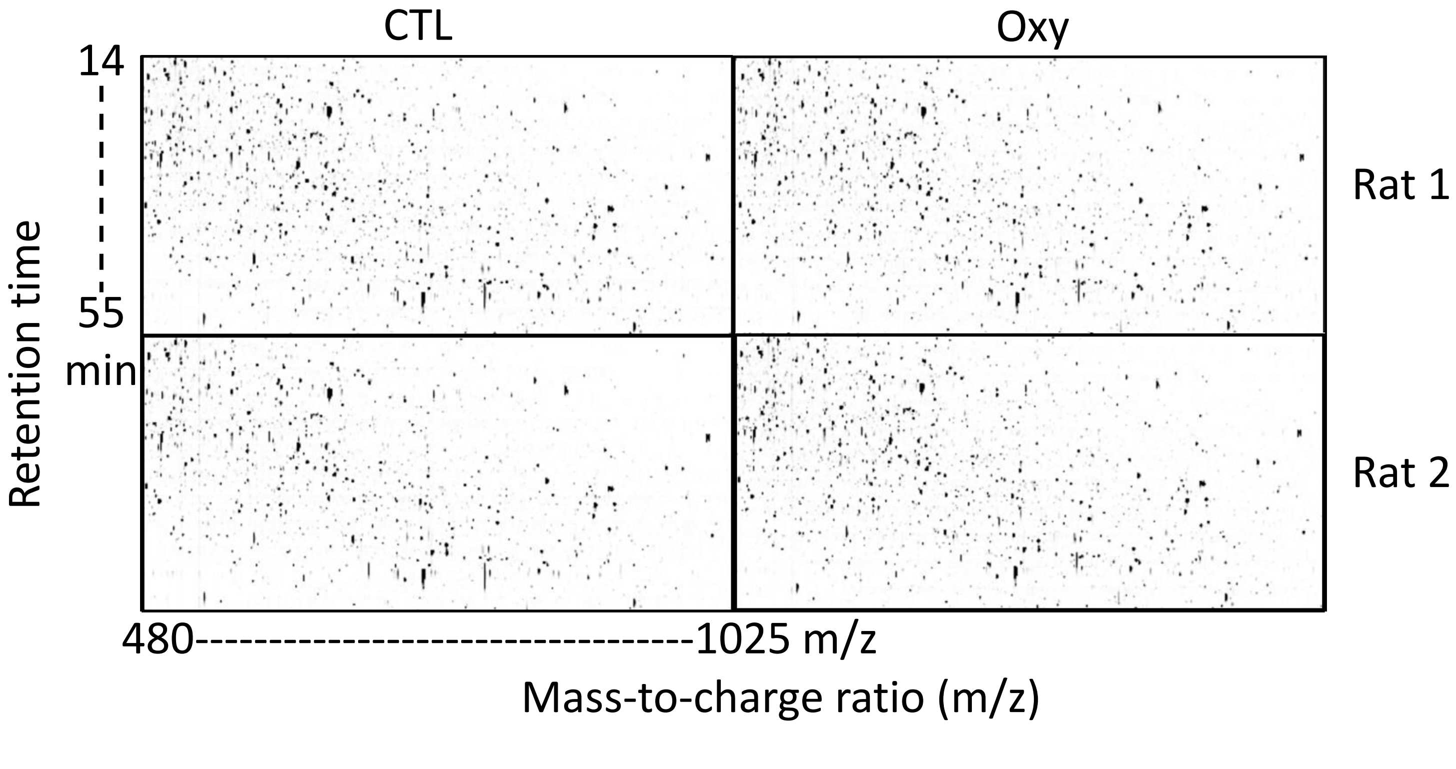

Supplementary Figure 1 – Example LC-MS data for control and oxidized rats after in vivo glycocapture for (A) N-glycosylated fraction and (B) Protease-released fraction (trypsin). Two-dimensional representation of the LC-MS data, produced using Msight, from two control (CTL) rats and two rats perfused with oxidation reagent (Oxy). Each spot represents a peptide ion. More peptides can be seen after oxidation in the N-glycosylated fraction, while the trypsin protease-released fraction shows abundant peptides even without oxidation.
